# A case of sleep paralysis in medical student: possibilities of poetry writing as self-therapy

**DOI:** 10.1192/j.eurpsy.2026.11729

**Published:** 2026-07-31

**Authors:** E. L. Nikolaev, F. V. Orlov, E. E. Nikolaev, H. Kumar

**Affiliations:** 1 Department of Social and Clinical Psychology; 2 Department of Psychiatry, Medical Psychology and Neurology; 3 Medical Faculty, I.N. Ulianov Chuvash State University, Cheboksary, Russian Federation

## Abstract

**Introduction:**

Sleep paralysis as a form of parasomnia is associated with poor sleep habits and perceived poor sleep quality (Duarte et al., 2023). Its prevalence in medical students ranges from 16% (Penn et al., 1981) up to 41% (Duarte et al., 2023) with 70% of females reporting experiencing sleep paralysis (Muzammil et al., 2023). Although this disorder does not necessarily testify to the presence of the disease, it requires an effective help.

**Objectives:**

In this case report, we present a case of sleep paralysis that appeared and developed in a young healthy student, who is studying in a medical university, and who is changing his attitude to this phenomenon due to rational psychotherapy and practice of poetry writing as self-therapy.

**Methods:**

We describe a 24-year-old male medical student, who has been under the observation of a psychotherapist because of periodic episodes of sleep paralysis.

**Results:**

Sleep disorders with the feeling of forcible retention when waking up and emergence of frightening visual images and death anxiety have been torturing the young man with different frequency since he was 18. At such moments he could not speak, shout, or move, his heart rate increased, and he was gripped by feelings of suffocation. Such state continued for 2-3 minutes. Those episodes appeared during intensive emotional stress associated with a high level of responsibility. Frequent disturbance of a sleep and rest regime provoked further bouts. We directed our diagnostic search at the exclusion of epilepsy and other paroxysmal states. The results of polysomnography did not show any specific changes. After the student heard from his psychiatrist that such states are not dangerous for health, the level of his anxiety lowered. He started analyzing his own experience of controlling and dealing with such states, worked at the techniques of discontinuing episodes of sleep paralysis. He began writing poems, in which he dissociated the problem from himself, which allowed him to avoid it and assure himself of his ability to cope with the problem, and to accept his actual state and his own fears.

**Conclusions:**

In this case, a medical student and his psychotherapist jointly tried out effective stress controlling strategies. Poetry writing, as self-therapy, helped him to adapt by acknowledging what is happening to him and to actively deal with stress even at critical moments. Knowledge of medicine helped him to form and accept an adequate attitude to impossibility of absolute control over his physical state.

**Disclosure of Interest:**

None Declared

